# Public Health, Hygiene and Bacteriology

**Published:** 1896-01

**Authors:** Ernest Wende

**Affiliations:** Health Commissioner of the City of Buffalo, N. Y.


### PUBLIC HEALTH, HYGIENE AND BACTERIOLOGY.

Conducted by ERNEST WENDE, M. D.,
Health Commissioner of the City of Buffalo, N. Y.

### NOVEMBER VITAL STATISTICS IN BUFFALO.

BY FRANKLIN C. GRAM, Registrar of Vital Statistics.

A DEATH rate of 12.33 per thousand for November, 1895, is a good contrast to 13.48 for the same month in 1894. This summarises the result of both months, although some of the details are interesting.

From communicable diseases there were 103 deaths, as against 115 in 1894. Ten died from senile debility, 3 from alcoholism, 16 from various kinds of cancer, 43 from diseases of the nervous system, 31 from the circulatory system, 57 from the respiratory system, 3 from appendicitis, and the remainder were distributed among various other causes, making a total of 346 deaths for the month. Of these, all but two were white, 198 were males and 148 were females. Twenty died by accident or violence, and 4 committed suicide. The nativities of these deceased were: from Buffalo, 175; other parts of the United States, 49; Germany, 58; Ireland, 26; Canada, 10; England, 11; Scotland, 3; Poland, 4; other foreigners, 6; unknown, 1.

I will present a similar table to the one given last month :

| | Tuberculosis. | Enteric Fever. | Diphtheria. | Diphtheritic Croup. | Membranous Croup. | Scarlet Fever. | Measles. | Pertussis. |
|---|---|---|---|---|---|---|---|---|
| Cases reported, Nov., 1895.. | 25 | 30 | 120 | 3 | 14 | 36 | 11 | 3 |
| Deaths, Nov., 1895......... | 33 | 13 | 26 | 1 | 12 | 0 | 1 | 3 |
| Cases reported, Nov., 1894.. | 0 | 31 | 102 | 5 | 14 | 112 | 21 | 2 |
| Deaths, Nov., 1894......... | 39 | 16 | 25 | 8 | 10 | 1 | 2 | 0 |
| Deaths, Nov., 1893 ........ | 44 | 4 | 12 | 13 | 0 | 19 | 1 | 7 |
| Deaths, Nov., 1892......... | 36 | 13 | 18 | 15 | 0 | 8 | 2 | 5 |
| Deaths, Nov., 1891......... | 46 | 22 | 12 | 24 | 0 | 14 | 0 | 0 |

There were 644 births, 244 marriages, 30 premature births and 37 still-births during November, 1895.

